# Less focus on symptom scales in psychiatric trials: it is time to ensure research equality between psychiatry and other medical specialities

**DOI:** 10.1016/j.lanepe.2024.100993

**Published:** 2024-07-02

**Authors:** Sophie Juul, Faiza Siddiqui, Pascal Faltermeier, Johanne Juul Petersen, Caroline Barkholt Kamp, Rikke Hermann Jakobsen, Lehana Thabane, Joanna Moncrieff, Mark Horowitz, Zainab Samaan, Michael Pascal Hengartner, Lawrence Mbuagbaw, Markus Harboe Olsen, Christian Gluud, Janus Christian Jakobsen

**Affiliations:** a Copenhagen Trial Unit, Centre for Clinical Intervention Research, The Capital Region, Copenhagen University Hospital – Rigshospitalet, Copenhagen, Denmark; b Stolpegaard Psychotherapy Centre, Mental Health Services, The Capital Region, Gentofte, Denmark; c Department of Psychology, University of Copenhagen, Copenhagen, Denmark; d Department of Regional Health Research, The Faculty of Health Sciences, University of Southern Denmark, Odense, Denmark; e Department of Health Research Methods, Evidence and Impact, McMaster University, Hamilton, Ontario, Canada; f St Joseph's Healthcare Hamilton, Hamilton, Ontario, Canada; g Division of Psychiatry, University College London, London, United Kingdom; h Research and Development Department, North East London NHS Foundation Trust (NELFT), London, United Kingdom; i Department of Psychiatry and Behavioural Neurosciences, McMaster University, Hamilton, Ontario, Canada; j Department of Applied Psychology, Zurich University of Applied Sciences, 8005, Zurich, Switzerland; k Department of Neuroanaesthesiology, The Neuroscience Centre, Copenhagen University Hospital – Rigshospitalet, Copenhagen, Denmark

## Comment

Every week, major randomised clinical trials involving thousands of participants are published in influential medical journals, yet it is uncommon for these trials to focus on psychiatric populations. For instance, we reviewed trials published in Lancet, Lancet Psychiatry, World Psychiatry, JAMA Psychiatry, American Journal of Psychiatry, Psychotherapy and Psychosomatics, and British Journal of Psychiatry from January 2022 until May 2024. We found no trials including a psychiatric population that randomised more than 1000 participants—a standard benchmark for a large trial.^1^ This scarcity presumably stems from the fact that many psychiatric trials primarily assess symptom scales as primary outcomes (Appendix 1), which typically leads to requiring smaller sample sizes. Trials within other medical specialities also often assess symptom scales (such as pain levels or functioning), but these outcomes are usually secondary.

Symptom scales, such as the Hamilton Depression Rating Scale (HDRS), among others, are routinely used in psychiatric research to evaluate symptoms. Most psychiatric treatments for both children and adults are primarily recommended based on their effectiveness in altering symptom scale scores.^2^^,^^3^ Although validated symptom scales are, of course, preferable over non-validated ones, employing symptom scales as primary outcomes presents several challenges (Box 1).

Box 1 **Challenges of using symptom scales as primary outcomes in psychiatric trials.**

First, symptom scales are typically assessed by the end of an intervention period. For most symptom scales, no evidence confirms that their scores are linked to long-term functional outcomes or prognosis.^4^ Long-term follow-up is crucial and urgently needed in psychiatric trials, as the patients involved often endure long-term conditions.

Second, it is crucial to determine a meaningful, quantifiable minimal important difference (MID) when using symptom scales as outcome measures rather than focusing solely on statistical significance. The MID represents the smallest benefit that patients consider valuable. Yet, for many psychiatric symptom scales, such as the commonly used HDRS, the MID remains uncertain and is a topic of extensive debate.^5^ Even in trials where anchor- or distribution-based methods have been used to quantify MIDs, the estimations often remain uncertain.

Third, significant psychometric challenges associated with symptom scales often complicate the interpretation of scores. Items may vary across scales, and reliability may be inconsistent.^6^ For example, the HDRS's measurement properties have been criticised, including debates over whether it should be treated as an ordinal rather than an interval scale. Issues like a decrease in insomnia potentially obscuring an increase in more critical symptoms like suicidality highlight the importance of revisiting how these scales are utilised and interpreted in clinical trials.^6^

Fourth, the subjective nature of symptom scales makes them susceptible to bias arising from the unblinding of patients and assessors by the adverse effects produced by active drugs or response expectancy from unblinded interventions such as psychotherapy.

Last, symptom scales are prone to bias from missing data, as they necessitate time- and resource-demanding responses to questionnaires or interviews. In a review of published trials assessing psychological interventions, we assessed missing data for 233 primary outcomes, of which 180 outcomes (77%) were assessed with a symptom scale with a mean proportion of missing data of 18.9% (Unpublished data). Such significant gaps in outcome data compromise the validity of a trial.

An effective approach to mitigate these challenges is to incorporate more ‘hard outcomes’ in psychiatric trials, such as instances of suicide or suicide attempts, hospital admissions, employment status, criminal convictions, reliance on social benefits, or educational completion.^7^ Collecting data on these outcomes is often easier and sometimes risk-free, especially in countries where registry data (like vital status) can be obtained seamlessly. Despite the advantages, hard outcomes are rarely chosen as primary measures in psychiatric research (Appendix 1), likely because of the considerably larger sample sizes needed. In contrast, large trials focusing on patient-important outcomes, such as all-cause mortality in somatic diseases, are frequently published in influential medical journals. Conducting large trials with hard outcomes in psychiatry should also be feasible. We have included examples of realistic sample size calculations in Appendix 2, showing that assessing hard outcomes in psychiatric trials leads to sample sizes corresponding to non-psychiatric trials published weekly in general medical journals. Furthermore, defining composite outcomes (for example, the proportion of participants with either a suicide, a suicide attempt, or a psychiatric acute hospitalisation) could decrease sample sizes even more.

Some may contend that psychiatric conditions are more complex than other conditions and, therefore, necessitate unique trial outcomes. However, like psychiatric conditions, physical conditions frequently result in symptoms (such as asthma symptoms or chest pain (angina) during physical exertion due to coronary heart disease), and the severity of these symptoms can also be measured using symptom scales. Moreover, patients with physical conditions often experience psychiatric symptoms (such as depression or anxiety). Despite these similarities, large non-psychiatric trials typically prioritise hard outcomes, such as death or myocardial infarction. Certain hard outcomes, like mortality, are equally significant for both psychiatric and non-psychiatric patients. Therefore, these outcomes should often be evaluated in both populations, and distinctions should not be made. It could be argued that the distinction between psychiatric and non-psychiatric conditions is not substantial, and symptoms are equally integral to the disease in both psychiatric and non-psychiatric patients. Psychiatric symptoms are evidently important to psychiatric patients, and symptom scales should be assessed in psychiatric trials. The question is if these scales should be used as primary outcomes and if sample size estimations should be quantified based on symptom scales. The severity of symptoms (such as angina in coronary heart disease trials) is often, and appropriately, considered a secondary outcome in non-psychiatric trials.

Knowledge about various psychiatric treatments is growing, but the overall methodological quality of these trials is hindered by an excessive dependence on symptom scales, which may not reflect outcomes most important to patients. Instead of conducting multiple small trials with poor methodology, it would be more rational to conduct fewer trials with larger sample sizes and adequate methodology. Such large trials will evidently be more expensive, but if it is possible to cover the costs of large trials in non-psychiatric research, it should be possible in psychiatric trials as well. Research in mental health is underfunded compared to other (physical) specialities.^8^ Research funders should allocate resources to large-scale psychiatric trials.

The scant data on hard outcomes is a major concern in psychiatric research,^9^ potentially obscuring the ‘true’ value of interventions. Core outcome sets can be developed to define essential patient-important outcomes for specific groups based on a consensus process, including relevant stakeholders like patients, relatives, clinicians, and researchers.^10^ By adopting such core outcome sets, researchers can measure and report outcomes that matter most to patients and healthcare decision-makers.

Prioritising patient relevance in outcome selection and measurement is crucial for enhancing the evidence base of psychiatric interventions and, ultimately, improving the mental health outcomes of psychiatric patients. Psychiatric patients deserve to have interventions backed by a quality of evidence comparable to that in other medical specialities. It is time to ensure equality between psychiatry and other medical specialities.

## Contributors

SJ and JCJ wrote up the first manuscript draft. FS, PF, JJP, CBK, RHJ, MH, MPH, JM, ZS, LT, LM, MHO, and CG significantly contributed to the writing and commented on the manuscript. All authors read and approved the final version of the manuscript.

## Declaration of interests

JM and MAH are collaborating investigators on the NHMRC- and MRFF-funded RELEASE and RELEASE + trials in Australia investigating hyperbolic tapering of antidepressants. MAH is a co-founder of Outro Health, a digital clinic which aims to help people who wish to stop no longer needed antidepressant medication in the US. MAH has received honoraria for lectures on deprescribing from NHS Trusts, Washington University and the University of Arizona. JM is a co-investigator on a National Institute of Health Research (NIHR) funded study exploring methods of antidepressant discontinuation (REDUCE) and the Chief Investigator on the RADAR trial of antipsychotic reduction and discontinuation funded by the NIHR. She collects royalties from three books on psychiatric drugs. ZS is a board member of the Canadian mood and anxiety disorders treatment guidelines. LM receives consulting fees from Astra Zeneca, Bayer, and WHO. All other authors declare that they have no competing interests.

## References

[1] Cappelleri J.C., Ioannidis J.P.A., Schmid C.H. (1996). Large trials vs meta-analysis of smaller trials: how do their results compare?. JAMA.

[2] National Institute for Health and Care Excellence (2021). Autism spectrum disorder in under 19s: support and management [CG170]. https://www.nice.org.uk/guidance/cg170/chapter/Recommendations.

[3] National Institute for Health and Care Excellence (2022). Depression in adults: treatment and management (CG222). https://www.nice.org.uk/guidance/ng222.

[4] McHugh C., Corderoy A., Ryan C., Hickie I., Large M. (2019). Association between suicidal ideation and suicide: meta-analyses of odds ratios, sensitivity, specificity and positive predictive value. BJPsych Open.

[5] Moncrieff J., Kirsch I. (2015). Empirically derived criteria cast doubt on the clinical significance of antidepressant-placebo differences. Contemp Clin Trials.

[6] Fried E.I., Flake J.K., Robinaugh D.J. (2022). Revisiting the theoretical and methodological foundations of depression measurement. Nature Rev Psychol.

[7] Grey P., Grey A., Bolland M.J. (2018). Outcomes, interventions and funding in randomised research published in high-impact journals. Trials.

[8] Woelbert E., White R., Lundell-Smith K., Grant J., Kemmer D., International Alliance of Mental Health Research Funders (2020). Inequities in mental health research funding. https://www.drugsandalcohol.ie/33429/1/The_Inequities_of_Mental_Health_Research.pdf.

[9] Papanikolaou P.N., Churchill R., Wahlbeck K., Ioannidis J.P. (2004). Safety reporting in randomized trials of mental health interventions. Am J Psychiatry.

[10] Williamson P.R., Altman D.G., Bagley H. (2017). The COMET handbook: version 1.0. Trials.

