# Supplementary material for: Less focus on symptom scales in psychiatric trials: it is time to ensure research equality between psychiatry and other medical specialities
Source: Lancet Reg Health Eur. 2024 Jul 2;43:100993. doi: 10.1016/j.lanepe.2024.100993 (PMC11269807; doi:10.1016/j.lanepe.2024.100993)
Supplement: Appendix 2 [file mmc2.docx]

**Appendix 2**

**Sample size estimations using hard outcomes - examples**

***Borderline personality disorder and self-harm:***

With an alpha (risk of type I errors) set at 5%, a beta (risk of type II errors) set at 80%, a relative risk reduction of 25% (based on the anticipated intervention effect in power calculations according to the GRADE Handbook [1]), and an estimated proportion of participants with an event in the control group set at 54% based on previous evidence [2], a total sample size of 462 participants will be needed.

***Schizophrenia and hospitalizations:***

With an alpha (risk of type I errors) set at 5%, a beta (risk of type II errors) set at 80%, a relative risk reduction of 25% [1], and an estimated proportion of participants with an event in the control group set at 29% based on previous evidence [3], a total sample size of 1,216 participants will be needed.

***Major depressive disorder and suicide attempts:***

With an alpha (risk of type I errors) set at 5%, a beta (risk of type II errors) set at 80%, a relative risk reduction of 25% [1], and an estimated proportion of participants with an event in the control group set at 24% based on previous evidence [4], a total sample size of 1,446 participants will be needed.

***Transdiagnostic participants with an index suicide attempt and non-fatal suicide reattempts***

With an alpha (risk of type I errors) set at 5%, a beta (risk of type II errors) set at 80%, a relative risk reduction of 25% [1], and an estimated proportion of participants with an event in the control group set at 28% based on previous evidence [5], a total sample size of 1,184 participants will be needed.

All sample size estimations are calculated using Stata version 17 [6].

**References**

1. Schünemann H, Brożek J, Guyatt G, Oxman A, The GRADE Working group. GRADE handbook for grading quality of evidence and strength of recommendations. 2013:Available from guidelinedevelopment.org/handbook.

2. Tate AE, Sahlin H, Liu S, Lu Y, Lundström S, Larsson H, et al. Borderline personality disorder: associations with psychiatric disorders, somatic illnesses, trauma, and adverse behaviors. Mol Psychiatry. 2022;27(5):2514-21.

3. Chen E, Bazargan-Hejazi S, Ani C, Hindman D, Pan D, Ebrahim G, et al. Schizophrenia hospitalization in the US 2005-2014: Examination of trends in demographics, length of stay, and cost. Med (Baltimore). 2021;100(15):e25206.

4. Dong M, Zeng L-N, Lu L, Li X-H, Ungvari GS, Ng CH, et al. Prevalence of suicide attempt in individuals with major depressive disorder: a meta-analysis of observational surveys. Psychological Medicine. 2019;49(10):1691-704.

5. Gibb SJ, Beautrais AL, Fergusson DM. Mortality and Further Suicidal Behaviour After an Index Suicide Attempt: a 10-Year Study. Australian & New Zealand Journal of Psychiatry. 2005;39(1-2):95-100.

6. StataCorp. Stata Statistical Software: Release 17. College Station, TX: StataCorp LLC.2021.
